# Safety of linezolid in patients with decreased renal function and trough monitoring: a systematic review and meta-analysis

**DOI:** 10.1186/s40360-022-00628-9

**Published:** 2022-11-30

**Authors:** Xiaoxi Liu, Mari Aoki, Sumika Osa, Chihiro Ito, Reika Saiki, Tomoya Nagai, Yuki Enoki, Kazuaki Taguchi, Kazuaki Matsumoto

**Affiliations:** Division of Pharmacodynamics, Keio University Faculty of Pharmacy, 1-5-30, Shibakoen, Minato-ku, Tokyo, 105-8512 Japan

**Keywords:** Linezolid, Hematological toxicity, Thrombocytopenia, Renal, Trough concentrations

## Abstract

**Background:**

Linezolid causes hematological toxicity, mostly thrombocytopenia, which leads to treatment discontinuation and failure. Recent studies revealed that during linezolid therapy, the incidence of treatment-related hematological toxicity is significantly higher in patients with decreased renal function (DRF) than in those with normal renal function. Linezolid monitoring is necessary due to the high frequency of hematological toxicity in patients with DRF and the relationship between blood concentration and safety. We performed a systematic review and meta-analysis to evaluate the safety correlation between DRF and trough monitoring.

**Methods:**

Articles published before June 24, 2022, on MEDLINE, Web of Sciences, Cochrane Register of Controlled Trials, and ClinicalTrials.gov were systematically analyzed. Odds ratios (ORs) and 95% confidence intervals (CIs) were calculated using the Mantel–Haenszel method and the variable effects model.

**Results:**

The incidence of hematological toxicity was significantly higher in patients with DRF than in those without DRF (OR = 2.37; *p* < 0.001). Subgroup analysis, performed according to hematotoxicity classification, including thrombocytopenia, anemia, and pancytopenia, revealed a significantly higher incidence of thrombocytopenia (OR = 2.45; *p* < 0.001) and anemia (OR = 2.31; *p* = 0.006) in patients with DRF than in those without; pancytopenia (OR = 1.41; *p* = 0.80) incidences were not significantly higher. Based on a systematic review, linezolid trough concentrations > 6–7 μg/mL may be associated with an increased incidence of thrombocytopenia. However, no confidential threshold values for the development of thrombocytopenia were found in the area under the concentration curve values for children or adults.

**Conclusion:**

We observed a high frequency of hematological toxicity during linezolid therapy in patients with DRF. To ensure safety, linezolid trough concentrations should be ≤6–7 μg/mL.

**Supplementary Information:**

The online version contains supplementary material available at 10.1186/s40360-022-00628-9.

## Introduction

Linezolid is an oxazolidinone antibiotic used to treat infectious diseases caused by drug-resistant gram-positive bacteria, such as methicillin-resistant *Staphylococcus aureus* and vancomycin-resistant *Enterococci*. Linezolid inhibits bacterial protein synthesis by binding to ribosomal RNA (30S and 50S ribosomal subunits) [1]. This unique mechanism prevents cross-resistance to existing antimicrobial agents of other classes [2]. However, the major treatment-related adverse event of linezolid therapy is hematological toxicity, mostly thrombocytopenia, which leads to treatment discontinuation and failure [3–5]. Generally, linezolid and its primary metabolites are excreted via non-renal (approximately 65%) and renal mechanisms [6]; therefore, dose adjustment is not required in patients with decreased renal function (DRF) [2, 7, 8]. However, recent studies have revealed that during linezolid therapy, the incidence of treatment-related hematological toxicity is significantly higher in patients with DRF than in those with normal renal function [9–13].

To avoid hematological toxicity, some studies have suggested that linezolid dose optimization based on its plasma concentration may be effective [14–16]. The pharmacokinetic (PK)/pharmacodynamic parameter of linezolid associated with effectiveness is the area under the concentration curve (AUC)/minimum inhibitory concentration [17, 18]. However, details of the concentrations and PK parameters associated with the safety evaluation of linezolid have not been clarified. In general, the trough concentration or AUC is used to evaluate the safety of antimicrobials. Although association of the trough concentration or AUC with the safety of linezolid has been frequently reported, it is unclear whether trough concentration or AUC is a suitable PK parameter for safety evaluation; furthermore, the appropriate range has yet to be determined. Systematic reviews and meta-analyses have recommended using vancomycin for safety monitoring cases with an AUC of 400–600 mg × h/L [19, 20]. However, no systematic review or meta-analysis has explored the concentrations or PK indices associated with linezolid safety.

Therefore, this meta-analysis aimed to determine whether hematological toxicity has a high incidence in patients with DRF. To avoid adverse events, we also performed a systematic review to evaluate linezolid’s monitoring parameters and ranges.

## Methods

### Search strategies

#### Search strategy for the evaluation of linezolid-associated hematotoxicity in patients with DRF

PubMed, Web of Sciences, Cochrane Register of Controlled Trials, and ClinicalTrials.gov databases were searched for relevant studies published before June 24, 2022. Two of four reviewers (MA, CI, RS, and TN) independently searched databases for literature using the following research terms: “linezolid,” “renal,” “kidney,” “thrombocytopenia,” “anemia,” “neutropenia,” “myelosuppression,” “leucopenia,” and “hematotoxicity.” The publication language was limited to English, and there was no restriction on the publication year. Duplicate articles were excluded.

#### Search strategy for the evaluation of linezolid monitoring and ranges

We similarly searched PubMed, Web of Sciences, Cochrane Register of Controlled Trials, and ClinicalTrials.gov databases for relevant studies published before June 24, 2022. Two of the four reviewers (MA, CI, RS, and TN) independently searched for literature using the following research terms: “linezolid,” “monitoring,” “area under the curve,” “trough,” and “therapeutic drug monitoring.” The publication language was limited to English, and there was no restriction on the publication year. Duplicate articles were excluded from the study.

### Study selection

#### Study selection for the evaluation of linezolid-associated hematotoxicity in patients with DRF

Two of the four reviewers (XL, MA, SO, and RS) independently screened the extracted literature. A study was considered eligible for evaluation in this meta-analysis provided that it met the following inclusion criteria: (1) the study included patients with and without DRF; (2) the study included patients who received linezolid treatment; and (3) the study revealed outcomes corresponding to hematotoxicity (thrombocytopenia, anemia, neutropenia, myelosuppression, and leukopenia). Studies that met the following criteria were excluded: (1) studies involving cells or animal models; and (2) case reports, case series, or reviews.

#### Study selection for the evaluation of linezolid monitoring and ranges

Two of the four reviewers (XL, MA, SO, and TN) independently screened the literature. A study was considered eligible for evaluation in this systematic review provided that it met the following inclusion criteria: (1) the study revealed the AUC or trough values of patients; (2) the study included patients who received treatment with linezolid; and (3) the study revealed the outcomes of thrombocytopenia.

### Data extraction

#### Data extraction for the evaluation of linezolid-associated hematotoxicity in patients with DRF

Two of the four reviewers (XL, SO, CI, and RS) independently extracted data from the studies. The study period, study design, country of the study, age and weight of the patients, definition of hematotoxicity, definition of DRF, and patients with and without DRF (patients with or without hematotoxicity were counted separately) were extracted according to the predefined eligibility criteria.

#### Data extraction for the evaluation of linezolid monitoring and ranges

Two of the four reviewers (XL, SO, CI, and RA) independently extracted data from the studies. The study period, study design, country of study, age of the patients, and AUC or trough values were extracted.

### Outcome analysis

#### Outcome analysis for the evaluation of linezolid-associated hematotoxicity in patients with DRF

The primary outcome was the incidence rate of hematotoxicity. The rate of hematotoxicity was defined according to each study’s definition. Subgroup analysis was performed according to the classification of hematotoxicity, including thrombocytopenia, anemia, pancytopenia, and myelosuppression.

#### Outcome analysis for the evaluation of linezolid monitoring and ranges

The primary outcome was the incidence of thrombocytopenia determined according to AUC_24_ (calculated by AUC_12_ if unavailable) and C_min_ (minimum blood plasma concentration) in children and adults.

### Assessment of the risk of bias

Two of the four reviewers (XL, SO, CI, and RA) independently assessed the risk of bias based on Cochrane Collaboration (Risk Of Bias In Non-Randomized Studies of Interventions, ROBINS-I) [21]. Discrepancies were resolved by discussion or consultation with the third reviewer (YE).

### Assessment of quality of evidence

The GRADE handbook was used to rate the grade quality of the meta-analysis [22]. GRADE specifies that the quality of the evidence can be classified into four categories according to the corresponding evaluation criteria: (1) high (⊕⊕⊕⊕); (2) moderate (⊕⊕⊕⊖); (3) low (⊕⊕⊖⊖); and (4) very low (⊕⊖⊖⊖).

### Analysis of the results and statistical analyses

The Review Manager for Windows (RevMan, Version 5.4, Copenhagen: The Nordic Cochrane Centre, The Collaboration, 2020) was used for data analysis and the preparation of forest plots. We used random-effects model for pooling study results. We calculated odds ratios (OR) with 95% confidence intervals (CIs) for discrete variables. To assess heterogeneity, *I*^2^ was calculated. Finally, funnel plots were constructed to assess potential publication bias.

### Protocol registration

The present study was not registered with Prospero or elsewhere.

## Results

### Search results

**Fig. 1:**
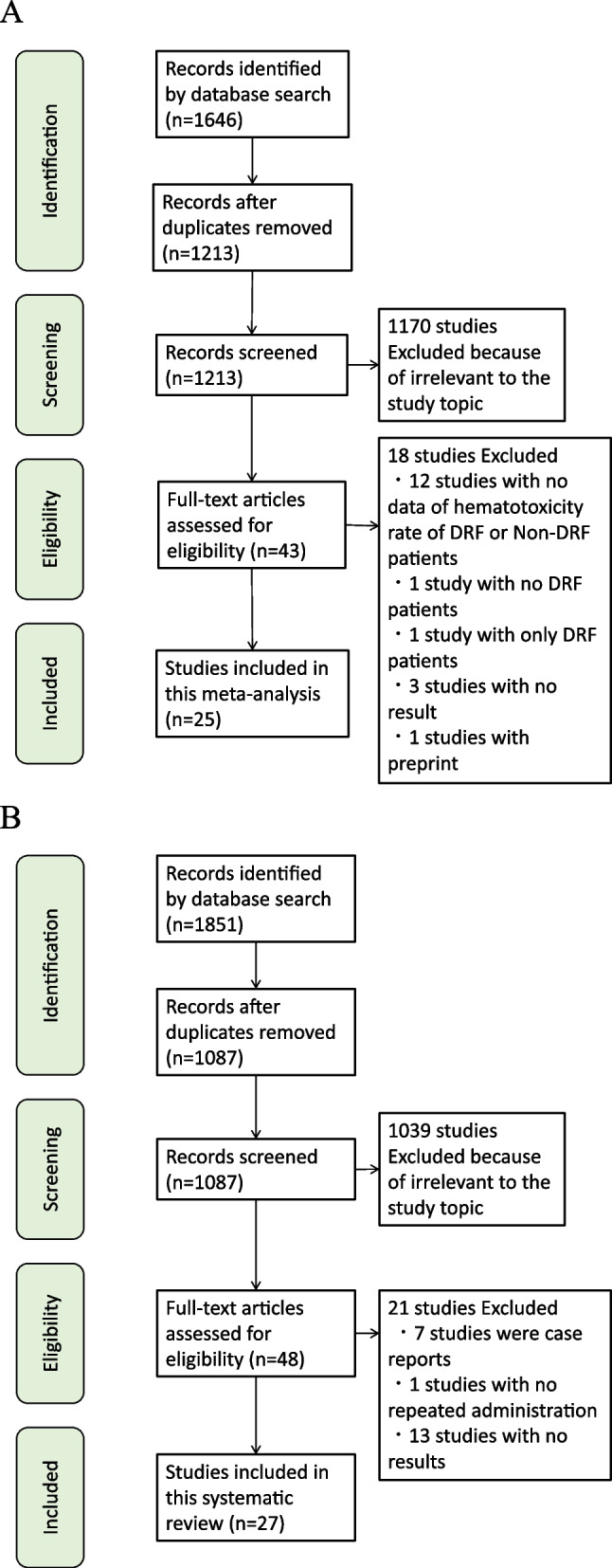
Flow chart of the study selection. Flow chart of **A** meta-analysis of hematotoxicity associated with linezolid, and **B** systematic review of hematotoxicity associated with the linezolid area under the concentration curve or C_min_ (minimum blood plasma concentration)

In the database search for the evaluation of linezolid-associated hematotoxicity, 1213 articles were screened after duplicates were extracted (Fig. 1A). Twenty-five articles [9–13, 23–42] were included for the evaluation of linezolid-associated hematotoxicity.

In the database search for the evaluation of linezolid monitoring and ranges, 1087 articles were screened after exclusion of duplicates (Fig. 1B). Twenty-seven articles [16, 23, 25, 43–66] were included in the evaluation of linezolid monitoring strategies.

### Characteristics

The characteristics of the 25 studies included in the meta-analysis for evaluating linezolid-associated hematotoxicity are shown in Table 1. These studies included 3831 patients, 1240 of whom had DRF. The definitions of DRF and hematotoxicity in each study are shown in Table 1. Most studies were conducted in Asian countries (16 of 25 studies). Twenty-three studies were retrospective, and two studies [25, 37] were prospective studies with a small number of cases conducted in Japan. Thrombocytopenia, anemia, pancytopenia, and reduction in neutrophils corresponded to hematotoxicity.

**Table 1:** Characteristics of the studies included in the meta-analysis

Study	Design of study	Country of study	Duration of study	Age of patients	No. of patients		Weight of patients	Definition of hematotoxicity	Definition of decreased renal function
					Decreased renal function	Non-decreased renal function			
Choi 2019 [9]	Retrospective longitudinal study	Korea	2005-2016	Mean: 63.4 ± 15.8	thrombocytopenia (45) non-thrombocytopenia (50)	thrombocytopenia (32) non-thrombocytopenia (137)	Mean: 58.4 ± 11.0	thrombocytopenia: platelet count < 100 × 10^3^ /mm^3^	CLcr < 30 mL/min
L. Crass 2019 [10]	Retrospective study	America	2007-2018	Mean: 54	thrombocytopenia (57) non-thrombocytopenia (76)	thrombocytopenia (35) non-thrombocytopenia (173)	Mean: 88	thrombocytopenia: platelet count < 112.5 × 10^3^ cells/μL	eGFR < 60 mL/min/1.73 m^2^
Dong 2014 [23]	Retrospective monocenter observational study	China	2008-2013	Mean: 58.6 ± 19.9	thrombocytopenia (8) non-thrombocytopenia (5)	thrombocytopenia (23) non-thrombocytopenia (34)	Mean: 64.5 ± 12.5	thrombocytopenia: decrease in platelet count of ≥25% and a final count of < 100 × 10^9^ /L	CLcr < 30 mL/min
Fujii 2014 [24]	Retrospective study	Japan	2011	Median: 64.0 ± 17.4 (21–86)	thrombocytopenia (6) non-thrombocytopenia (10)	thrombocytopenia (31) non-thrombocytopenia (44)	Median: 56.6 ± 10.0 (37.0–84.5)	thrombocytopenia: ≥ 30% decrease in platelet count from the baseline value	eGFR < 30 ml/min/1.73 m^2^
Giunio-Zorkin 2019 [11]	Retrospective observational cohort study	Canada	2013-2017	Mean: 58 ± 17 (Thrombocytopenia patients) 49 ± 22 (Non-thrombocytopenia patients)	thrombocytopenia (11) non-thrombocytopenia (27)	thrombocytopenia (7) non-thrombocytopenia (57)	Mean: 69 ± 16 (Thrombocytopenia patients) 65 ± 21 (Non-thrombocytopenia patients)	thrombocytopenia: platelet count < 100 × 10^9^ /L or ≥ 50% reduction from baseline	serum creatinine > 90 μmol/L for females; > 100 μmol/L for males
Hiraki 2012 [25]	Prospective study	Japan	―	Mean: 64.6 ± 10.9	thrombocytopenia (3) non-thrombocytopenia (0)	thrombocytopenia (2) non-thrombocytopenia (3)	Mean:54.9 ± 10.7	thrombocytopenia: a decrease in the PLT count of ≥50%	CLcr < 60 ml/min
Hirano 2014 [26]	Retrospective study	Japan	2010-2012	Mean: 69.0 ± 11.5 (Thrombocytopenia patients) 62.4 ± 17.2 (Non-thrombocytopenia patients)	Thrombocytopenia (7) non-thrombocytopenia (3)	thrombocytopenia (22) non-thrombocytopenia (43)	Mean: 57.5 ± 11.9 (Thrombocytopenia patients) 55.2 ± 11.5 (Non-thrombocytopenia patients)	thrombocytopenia: a decrease in the patient’s platelet count to < 10 × 10^4^ /μL or a reduction of ≥30% from their baseline value	CLcr < 30 mL/min
Han 2022 [34]	Retrospective study	China	2015-2021	Mean: 69.67 ± 16.39	Thrombocytopenia (39) non-thrombocytopenia (88)	Thrombocytopenia (34) non-thrombocytopenia (159)	―	thrombocytopenia: platelet count of < 100 × 10^9^ /L	CLcr < 60 mL/min
Hsu 2022 [35]	Retrospective cohort study	Taiwan	2019	Mean: 71.0 ± 16.1 (Thrombocytopenia patients) 66.7 ± 15.2 (Non-thrombocytopenia patients)	Thrombocytopenia (21) non-thrombocytopenia (23)	Thrombocytopenia (31) non-thrombocytopenia (23)	―	thrombocytopenia: platelet count of < 100 × 10^9^ /L or a decrease of in 25% or more from the baseline	CLcr < 60 mL/min
Jones 2015 [27]	Retrospective single-center cohort study	America	2007-2012	Median: 6 (1–13) (Thrombocytopenia patients) 9 (3.1–14.7) (Non-thrombocytopenia patients)	thrombocytopenia (21) non-thrombocytopenia (16)	thrombocytopenia (27) non-thrombocytopenia (98)	Median: 23.8 (7.4–44.7) (Thrombocytopenia patients) 27.3 (13.8–47.3) (Non-thrombocytopenia patients)	thrombocytopenia: platelet count of < 100,000 platelets/mm^3^ or a reduction of ≥30% from the baseline platelet count	CLcr < 60 ml/min/1.73 m^2^
Kim 2019 [12]	Retrospective study	Korea	2005-2015	Mean: 70.6 ± 13.3 (Thrombocytopenia patients) 69.1 ± 10.5 (Non-thrombocytopenia patients)	Thrombocytopenia (13) non-thrombocytopenia (9)	thrombocytopenia (16) non-thrombocytopenia (22)	Mean: 55.2 ± 9.5 (Thrombocytopenia patients) 57.3 ± 10.6 (Non-thrombocytopenia patients)	thrombocytopenia: platelet count of < 150 × 10^9^ /L or a decrease of at least 50% from the baseline	Chronic kidney disease
Kawasuji 2021 [36]	Monocentric, retrospective, observational study	Japan	2013-2019	Median: 71 (58.5–78)	thrombocytopenia (22) non-thrombocytopenia (13)	thrombocytopenia (26) non-thrombocytopenia (57)	Median: 57.1 (48.0–64.2)	thrombocytopenia: platelet count of < 112.5 × 10^3^/μL or a decrease of in 25% or more from the baseline	CL_CRC-G_ ≤ 60 mL/min
Komatsu 2022 [37]	Prospective interventional study	Japan	2017-2020	Median: 68(61-75) (Patients within therapeutic range) 70(63-74) (Patients above therapeutic range)	thrombocytopenia (3) non-thrombocytopenia (4)	thrombocytopenia (10) non-thrombocytopenia (20)	Median: 54.0(45.7-64.6) (Patients within therapeutic range) 67.4(57.8-75.9) (Patients above therapeutic range)	thrombocytopenia: decrease of in 30% or more from the baseline	CLcr < 50 mL/min
Lima 2020 [13]	Retrospective cohort study	Brazil	2015-2017	Median: 67 (34–101) (Thrombocytopenia patients) 61 (18–90) (Non-thrombocytopenia patients)	thrombocytopenia (6) non-thrombocytopenia (16)	thrombocytopenia (4) non-thrombocytopenia (34)	Median: 65.5 (51.1–81) (Thrombocytopenia patients) 68 (34–160) (Non-thrombocytopenia patients)	thrombocytopenia: decrease in platelet count of ≥20% from the baseline level and a final count of < 100 × 10^3^ /mm^3^	CLcr < 30 mL/min
Lin 2006 [28]	Retrospective case-control study	Taiwan	2002-2004	Mean: 53.6 ± 19.4 (renal insufficiency patients) 58.2 ± 21.0 (non-renal insufficiency patients)	anemia (6) non-anemia (11) thrombocytopenia (11) non-thrombocytopenia (6) pancytopenia (0) non-pancytopenia (17)	anemia (17) non-anemia (28) thrombocytopenia (16) non-thrombocytopenia (29) pancytopenia (4) non-pancytopenia (41)	―	anaemia: haemoglobin < 10 mg/dL thrombocytopenia: platelet count < 100 × 10^9^ platelets/L pancytopenia: ANC < 500 × 10^6^ /L	serum creatinine ≥1.3 mg/dL for women and ≥ 1.5 mg/dL for men
Moraza 2015 [29]	Retrospective observational study	Spain	―	Median: 73 (23-91)	hematological toxicity (2) non-hematological toxicity (1)	hematological toxicity (14) non- hematological toxicity (21)	Median: 68.5(41.3-103)	hepatotoxicity: HR ≥ 25% PR ≥ 25% and/or NR ≥ 50% HR: rate of reduction in the level of hemoglobin; PR: rate of reduction in platelet count; NR: rate of reduction in neutrophil count.	CLcr < 30 ml/min
Maray 2022 [38]	Retrospective study	spain	2001-2012	Median: 61.36 (51.39–71.73)	thrombocytopenia (14) non-thrombocytopenia (24)	thrombocytopenia (49) non-thrombocytopenia (233)	Median: 86.20 (70.00–103.60)	thrombocytopenia: decrease of at least 50% from the baseline platelet count	Acute Kidney Injury (AKIN) II or greater
Plachouras 2006 [30]	Retrospective study	Greece	2004-2005	Mean: 61.4 ± 13.5	myelosuppression (4) non-myelosuppression (2)	Myelosuppression (7) non-myelosuppression (12)	―	myelosuppression: hematocrit decreased to 30% or the platelet count decreased to < 140 × 10^9^ platelets/L	Chronic renal failure
Qin 2021 [39]	Retrospective study	China	2014-2020	Median: 63.0 (45.3 ~ 71.3) (Anemia patients) 55.0 (37.0 ~ 66.0) (Non-anemia patients)	anemia (11) non-anemia (45)	anemia (21) non-anemia (221)	Median: 60.0 (55.0-66.0) (Anemia patients) 62.8 (55.0-71.3) (Non-anemia patients)	anemia: Hb count to 75% of the baseline value	eGFR < 60 ml/(min·1.73m^2^)
Rabon 2018 [31]	Retrospective study	America	2014-2016	Median: 59 (43-66) (Thrombocytopenia patients) 53 (36-64) (non-thrombocytopenia patients)	thrombocytopenia (21) non-thrombocytopenia (22)	thrombocytopenia (36) non-thrombocytopenia (80)	Median: 78 (62-92) (Thrombocytopenia patients) 83 (67-98) (non-thrombocytopenia patients)	thrombocytopenia: platelet count < 150 × 10^9^ /L or platelet count < 75% of 112.5 × 10^9^ /L or a reduction of ≥50% from baseline platelet count	eGFR < 30 mL/min/1.73 m^2^
Sato 2020 [40]	Retrospective cohort study	Japan	2011-2014	Mean: 57.4 ± 23.3	thrombocytopenia (3) non-thrombocytopenia (5)	thrombocytopenia (14) non-thrombocytopenia (15)	Mean: 55.1 ± 20.8 (Thrombocytopenia patients) 53.4 ± 24.5 (non-thrombocytopenia patients)	thrombocytopenia: platelet count of < 100 × 10^9^ /L or at least a decrease of in 50% more from the baseline	Chronic kidney disease
Takahashi 2011 [32]	Retrospective study	Japan	2007-2009	Mean: 60.7 ± 19.9 (Thrombocytopenia patients) 56.3 ± 20.2 (non-thrombocytopenia patients)	thrombocytopenia (74) non-thrombocytopenia (77)	thrombocytopenia (54) non-thrombocytopenia (126)	Mean: 54.1 ± 13.6 (Thrombocytopenia patients) 55.0 ± 14.1 (non-thrombocytopenia patients)	thrombocytopenia: ≥ 10 × 10^4^ cells/mm^3^ decrease from the baseline or ≥ 30% reduction from the baseline	CLcr < 50 mL/min
Thirot 2021 [41]	Retrospective study	Belgian	2016	Median: 65 (21–95)	thrombocytopenia (30) non-thrombocytopenia (84)	thrombocytopenia (13) non-thrombocytopenia (101)	Median: 76 (34–178)	thrombocytopenia: platelet count of < 150 × 10^9^ /L and ≥ 30% reduction from the baseline	CLcr < 60 mL/min
Wu 2006 [33]	Retrospective case-control study	Taiwan	2002-2004	Mean: 72.1 ± 10.8 (renal insufficiency patients) 56.8 ± 20.4 (non-renal insufficiency patients)	anemia (20) non-anemia (8) thrombocytopenia (22) non-thrombocytopenia (6) pancytopenia (6) non-pancytopenia (22)	anemia (23) non-anemia (40) thrombocytopenia (27) non-thrombocytopenia (36) pancytopenia (4) non-pancytopenia (59)	―	thrombocytopenia: platelet count < 100 × 10^9^ platelets/L anemia: hemoglobin level < 10 mg/dL pancytopenia: ANC < 500 × 10^6^ neutrophils/L	patients with end-stage renal disease (ESRD)
Wu 2022 [42]	Retrospective study	Taiwan	2018-2019	Median: 62 [16–99]	anemia (10) non-anemia (32) thrombocytopenia (24) non-thrombocytopenia (18)	anemia (5) non-anemia (35) thrombocytopenia (18) non-thrombocytopenia (22)	Median: 64 [40–110]	thrombocytopenia: PLT < 125 × 10^9^ cells/L and a decrease ≥25% of PLT from baseline levels anemia: a reduction of ≥25% of Hb compared with the baseline.	CLcr < 60 mL/min

The characteristics of the 27 systematically reviewed studies are shown in Tables 2, 3, 4 and 5. Tables 2 and 3 show studies that evaluated the incidence of thrombocytopenia associated with AUC values in children and adults, respectively. In the analysis of AUC values associated with thrombocytopenia, two studies involved children (Table 2), and 15 studies involved adults (Table 3). A total of 230 patients (including eight children) were included in the analysis. All studies analyzing AUC values associated with thrombocytopenia in children were prospective studies. Of the 15 adult studies, two were retrospective studies, while 12 were prospective studies, on the analysis of AUC values associated with thrombocytopenia in adults. The National Institute of Allergy and Infectious Diseases (NIAID) study in 2018 was a clinical trial.

**Table 2:** Characteristics of the studies included in the systematic review about AUC (children)

Study	Design of study	Country of study	Duration of therapy (days)	Age of children	No. of children	AUC (mg・h/L) of children	
						Thrombocytopenia	Non-thrombocytopenia
Kosaka 2009 [43]	Prospective study	Japan	Mean: 47.5 ± 48.4	Mean: 1.2 ± 0.8	4 (0/4)	―	AUC_24_ 207.6, 361.2^a^
Matsumoto 2014 [44]	Prospective observational study	Japan	Mean: 17.8 ± 7.0	Mean: 6.4 ± 3.2	5 (1/4)	AUC_24_ 180.5^b^	AUC_24_ 116.5, 161.1, 186.4, 231.2

^a^Only 2 of 4 cases’ AUC was calculated

^b^Concomitantly used methotrexate

**Table 3:** Characteristics of the studies included in the systematic review AUC (adults)

Study	Design of study	Country of study	Duration of therapy (days)	Age of patients	No. of patients	AUC (mg・h/L) of patients	
						Thrombocytopenia	Non-thrombocytopenia
NIAID 2018 [47]	Clinical Trial	Brazil, America	7	18-65	10 (0/10)	―	AUC_24_ Median: 232.9
Alffenaar 2010 [45]	Prospective pharmacokinetic study	Netherlands	Median: 56	Median: 28	8 (0/8)	―	AUC_12_ median:145.8 (AUC_24_ median:291.6)
Alffenaar 2010 [65]	Prospective pharmacokinetic study	Netherlands	Median: 49	Median: 28	12 (0/12)	―	AUC_12_ Median: 123.8 (AUC_24_ median:247.6)
Beer 2007 [46]	Prospective study	Austria	> 7	Mean: 49.2 ± 19.5	5 (0/5)	―	AUC_12_ Mean: 86.5 ± 44.5 (AUC_24_ mean:173)
Bhalodi 2013 [48]	Prospective pharmacokinetic study	America	2.5	Mean: 42.2 ± 12.2	20 (0/20)	―	AUC_12_ Mean: 119.8 ± 46.24 (AUC_24_ mean:239.6)
Boak 2014 [49]	Prospective observational study	America	Mean: 22	Mean: 54.0 (Thrombocytopenia patients) 60.5 (Non-thrombocytopenia patients)	38 (10/28)	AUC_24_ Mean: 243	AUC_24_ Mean: 213
Blackman 2021 [66]	prospective study	America	Mean: 4.6 ± 2.8	59.6: ±13.0	11(2/11)	AUC_24_: 345.8, 175.0^a^.	AUC_24:_ 137.9, 233.6, 142.0, 144.0, 321.9, 191.6^a^, 142.6^a^, 126.3^a^, 328.3^a^
Conte 2002 [50]	Prospective study	America	2.5	Mean: 30 ± 5	25 (0/25)	―	AUC24 Mean: 204.2
Eslam 2014 [51]	Prospective study	Austria	≧3	59-81	10 (0/10)	―	AUC_24_ Mean: 164.5 ± 62.1
Gee 2001 [52]	Prospective study	United Kingdom	2.5	Mean: 29.6	6 (0/6)	―	AUC_12_ Mean: 107.5 ± 40.6 (AUC_24_ mean:215)
Luque 2014 [53]	Prospective pharmacokinetic study	Spain	> 3	Mean: 51.9 ± 10.3	11 (0/11)	―	AUC_12_ Median: 47.6 (AUC_24_ median:95.2)
Myrianthefs 2006 [54]	Prospective study	Greece	≧2	Mean: 58.7 ± 17.3	14 (0/14)	―	AUC_12_ Mean: 128.7 ± 83.9 (AUC_24_ mean:257.4)
Pea 2012 [16]	Retrospective observational study	Italy	Median: 63	Mean: 49.9 ± 15.2	35 (16/19)	AUC_24_ 280.74 (50% probability) 343.02 (95% probability)	―
Swoboda 2010 [55]	Retrospective study	Germany	2-4	Mean: 57.2 ± 11.9 (septic patients on extended dialysis) 68.6 ± 4.2 (septic patients without dialysis)	15 (0/15)	―	AUC_24_ Mean:115.2 ± 70.6 (with dialysis) 123.5 ± 124.4 (without dialysis)
Traunmüller 2010 [56]	Prospective study	Austria	―	60-67	3 (0/3)	―	AUC_24_ Median: 229.4

^a^Three times daily 600 mg linezolid was administered

**Table 4:** Characteristics of the studies included in the systematic review about C_min_ (children)

Study	Design of study	Country of study	Duration of therapy (days)	Age of children	No. of children	C_min_ (μg/ml) of children	
						Thrombocytopenia	Non-thrombocytopenia
Cojutti 2015 [57]	Retrospective study	Italy	Group1 Median: 15.7 Group2 Median: 11	Group1 Mean: 4.9 ± 2.8 Group2 Mean: 14.9 ± 1.3	23 (8/15)	Median: 7.17	―
Kosaka 2009 [43]	Prospective study	Japan	Mean: 47.5 ± 48.4	Mean: 1.2 ± 0.8	4 (0/4)	―	0.1, 1.9, 2.7, 3.5, 4.1^a^
Matsumoto 2014 [44]	Prospective observational study	Japan	Mean: 17.8 ± 7.0	Mean: 6.4 ± 3.2	5 (1/4)	4.7^b^	1.4, 1.8, 4.4, 4.6

^a^One patient’ C_min_ values were measured both after administered intravenously and orally

^b^Concomitantly used methotrexate

**Table 5:** Characteristics of the studies included in the systematic review about C_min_ (adults)

Study	Design of study	Country of study	Duration of therapy (days)	Age of patients	No. of patients	C_min_ (μg/ml) of patients	
						Thrombocytopenia	Non-thrombocytopenia
Alffenaar 2010 [45]	Prospective pharmacokinetic study	Netherlands	Median: 56	Median: 28	8 (0/8)	―	Median: 5.8
Alffenaar 2010 [65]	Prospective pharmacokinetic study	Netherlands	Median: 49	Median: 28	12 (0/12)	―	Median: 4.4
Beer 2007 [46]	Prospective study	Austria	> 7	Mean: 49.2 ± 19.5	5 (0/5)	―	Mean: 1.94 ± 1.69
Cojutti 2019 [58]	Prospective interventional study	Italy	Median: 19-54	Median: 62	61 (9/52)	4.28, 6.81, 7.32, 9.9, 10.0, 11.43, 14.83, 16.43, 27.88	―
Conte 2002 [50]	Prospective study	America	2.5	Mean: 30 ± 5	25 (0/25)	―	Mean: 0.2 ± 0.2
Dong 2014 [23]	Retrospective observational study	China	Mean: 11.3 ± 5.7	Mean: 58.6 ± 19.9	70 (31/39)	Median: 8.81	Median: 2.88
Fang 2020 [59]	Prospective observational study	China	Mean: 10.0 ± 5.3	Mean: 69.6 ± 13.8	84 (18/66)	7.85 (50% probability) 10.55 (95% probability)	―
Hiraki 2012 [25]	Prospective study	Japan	Mean: 14.3 ± 11.0	Mean: 64.6 ± 10.9	8 (5/3)	higher than 22.1 μg/ml (50% hazard ratio)	―
Luque 2014 [53]	Prospective pharmacokinetic study	Spain	> 3	Mean: 51.9 ± 10.3	11 (0/11)	―	<0.2-2
Luque 2019 [60]	Retrospective observational study	Spain	Median: 9 (cases with liver cirrhosis) 11 (controls)	Median: 67.5 (cases with liver cirrhosis) 61.5 (controls)	52 (21/31)	Median: 20.4	Median: 4.9
Matsumoto 2014 [61]	Prospective observational study	Japan	Mean: 12.9 ± 6.4	Mean: 70.6 ± 10.3	44 (35/9)	8.2 (50% probability)	―
Morata 2013 [62]	Retrospective study	Spain	3-10	Mean: 60.8 ± 17.4 (Cmin<2 mg/L) 66.8 ± 16.6 (Cmin>2 mg/L)	78 (6/72)	Median: 12.9	Median: 4.2
Myrianthefs 2006 [54]	Prospective study	Greece	≧2	Mean: 58.7 ± 17.3	14 (0/14)	―	Mean: 5.6 ± 5.0
Nukui 2013 [63]	Prospective observational study	Japan	Median: 12	Median: 46	30 (17/13)	day3: 13.4, day7: 15.3, day14: 15.2 threshold value > 7.5	day3: 4.3, day7: 3.8, day14: 5.0
Pea 2012 [16]	Retrospective observational study	Italy	Median: 63	Mean: 49.9 ± 15.2	35 (16/19)	6.53 (50% probability) 9.96 (95% probability)	―
Swoboda 2010 [55]	Retrospective study	Germany	2-4	Mean: 57.2 ± 11.9 (septic patients on extended dialysis) 68.6 ± 4.2 (septic patients without dialysis)	15 (0/15)	―	Median: 1.0 (with dialysis) 0.5 (without dialysis)
Tsuji 2011 [64]	Prospective observational study	Japan	Mean: 12.0 ± 10.2	Mean: 66.9 ± 6.6	12 (2/10)	mean:35.4 ± 13.5 (Grade2) mean:67.7 ± 17.1 (Grade4)	―

Tables 4 and 5 list studies that evaluated the incidence of thrombocytopenia associated with C_min_ in children and adults, respectively. In the analysis of C_min_ associated with thrombocytopenia, three studies included children (Table 4), and 17 studies included adults (Table 5). Two of the three studies were prospective in the analysis of C_min_ associated with thrombocytopenia in children. Twelve of the 14 studies were prospective studies that analyzed C_min_ associated with thrombocytopenia in adults.

### Outcome analysis for the evaluation of linezolid-associated hematotoxicity in patients with DRF

**Fig. 2:**
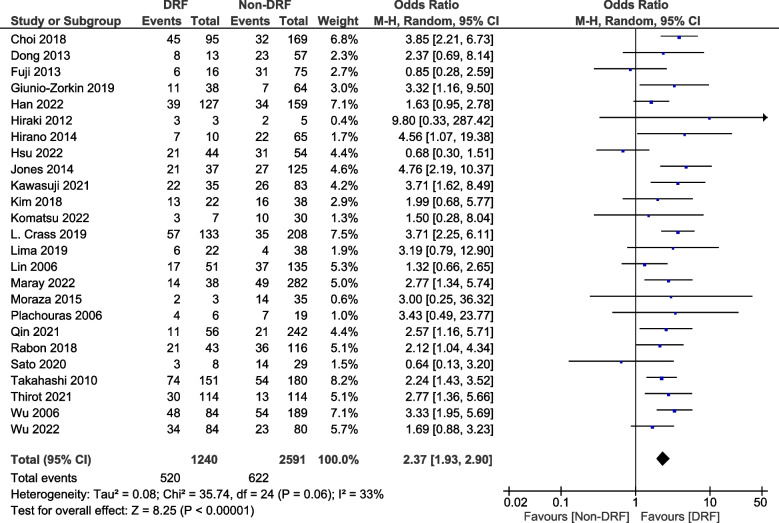
Forest plot of the hematotoxicity associated with linezolid treatment with or without decreased renal function. Vertical line indicates no significant differences between the groups. Diamond shapes and horizontal lines indicate odds ratios and 95% confidence intervals, respectively. Squares indicate point estimates and the size of each square indicates the weight of each study

Twenty-three retrospective studies and two prospective studies with 1240 patients with DRF and 2591 patients without DRF were enrolled in the meta-analysis. Compared with patients without DRF, patients with DRF had a significantly higher incidence of hematotoxicity (OR = 2.37; 95% CI: 1.93–2.90; *p* < 0.001; *I*^2^ = 33%) (Fig. 2).

**Fig. 3:**
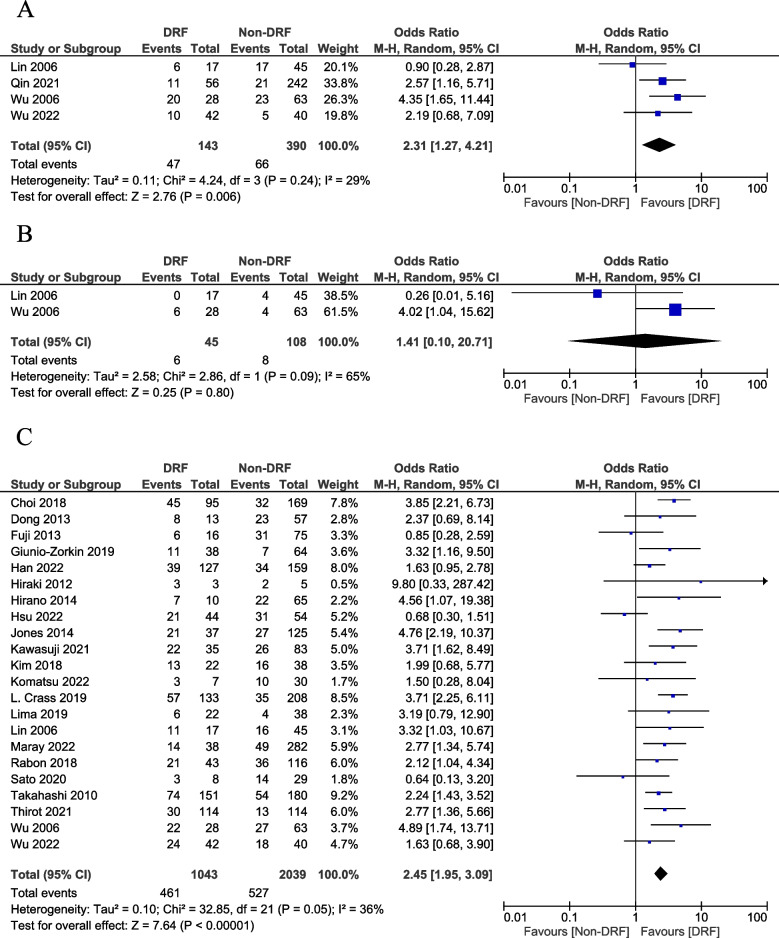
Forest plot of the subgroup analysis of the hematotoxicity classification associated with linezolid treatment with or without decreased renal function. Vertical line indicates no significant differences between the groups. Diamond shapes and horizontal lines indicate odds ratios and 95% confidence intervals, respectively. Squares indicate point estimates and the size of each square indicates the weight of each study. Subgroup analysis of **A** anemia; **B** pancytopenia; and **C** thrombocytopenia

We also conducted a subgroup analysis based on the classification of hematotoxicity. The incidences of thrombocytopenia (OR = 2.45; 95% CI: 1.95–3.09; *p* < 0.001; *I*^2^ = 36%) and anemia (OR = 2.31; 95% CI: 1.27–4.21; *p* = 0.006; *I*^*2*^ = 29%) were significantly higher in patients with DRF than in those without DRF (Fig. 3A and C). However, no significant differences were observed in the incidence of pancytopenia (OR = 1.41; 95% CI: 0.10–20.72; *p* = 0.80, *I*^2^ = 65%) in patients with and without DRF (Fig. 3B).

### Outcome analysis for AUC values and the incidence of thrombocytopenia

No confidential threshold values for the development of thrombocytopenia were found in AUC values for children or adults (Tables 2 and 3). Only four studies reported the AUC values for patients with thrombocytopenia, and the values were 180.5 [44] 243 [49], 280.74 [16], and 175.0 or 345.8 [66] mg × h/L. Thrombocytopenia did not occur when the mean or median AUC_24_ (calculated by AUC_12_ if it was not available) was within 95.2–328.3 mg × h/L in adults (Table 3).

### Outcome analysis for C_min_ and the incidence of thrombocytopenia

Twelve studies reported the incidence of thrombocytopenia. In the analysis for children, two studies revealed the incidence of thrombocytopenia, and the C_min_ values of thrombocytopenia and non-thrombocytopenia were 4.7–7.17 and 0.1–4.6 μg/mL, respectively. One patient with a C_min_ value of 4.7 μg/mL received high-dose methotrexate in combination treatment. In the adult analysis, 10 studies revealed the incidence of thrombocytopenia, and the C_min_ values of thrombocytopenia and non-thrombocytopenia were 4.28–67.7 and 0.2–5.8 μg/mL, respectively. In seven studies, C_min_ for patients without thrombocytopenia was not determined. Except for a C_min_ of 4.28 μg/mL, thrombocytopenia occurred at C_min_ values of > 6–7 μg/mL.

### Publication bias

**Fig. 4:**
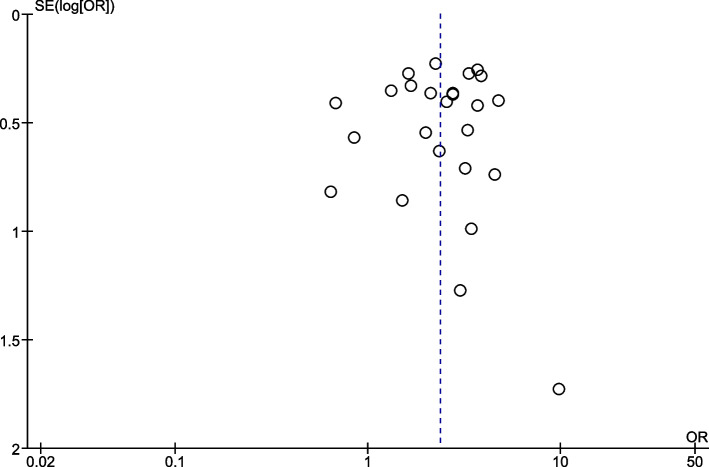
Publication bias plot of 16 trials of linezolid-associated hematotoxicity in patients with decreased renal function

Funnel plots of the incidence of hematotoxicity are shown in Fig. 4. The funnel plots were symmetric and did not suggest the presence of publication bias in favor of a positive study for all outcomes.

### Assessment of the risk of bias

The results of the assessment of the risk of bias are presented in Figs. S1 and S2. A high risk of confounding bias was found in the study by Hiraki et al. [25]. Information regarding selection bias was unavailable for most studies; few studies identified bias issues. No problems in intervention bias were identified, and moderate missing data bias was identified in the study by Choi 2019. Three studies [30, 33, 40] had a moderate risk of measurement of outcome bias. No information was available for deviation from the intended intervention and reporting biases.

### Quality of the evidence

The results of the quality evaluation according to the GRADE guideline are shown in Table 6. This meta-analysis consisted primarily of observational studies, so there was a low initial rating. Some problems in the risk of bias downgraded the quality of evidence by one level, while a large magnitude of effect upgraded the quality of evidence by one level. The low final grade of the evidence shows that our confidence in the effect estimate is limited.

**Table 6:** GRADE assessment of meta-analysis

Factor	Consequence
Study design	⊕⊕⊖⊖
Risk of bias	↓^a^
Inconsistency of results	→
Indirectness of evidence	→
Imprecision	→
Publication bias	→
Large magnitude of effect	↑^b^
All plausible confounding would reduce the demonstrated effect or increase the effect if no effect was observed	→
Dose-response gradient	→
GRADE quality	⊕⊕⊖⊖

GRADE assessment criteria; ⊕⊕⊕⊕:high, ⊕⊕⊕⊖:moderate, ⊕⊕⊖⊖:low, ⊕⊖⊖⊖:very low

^a^Downgrade

^b^Upgrade

## Discussion

In this meta-analysis of retrospective and prospective studies, the incidence of hematotoxicity was significantly higher in patients with DRF than in those without. In addition, subgroup analysis revealed a significant difference in the incidence of thrombocytopenia and anemia, but there was no significant difference in the incidence of pancytopenia (Fig. 3A–C). These results suggest that linezolid should be cautiously administered in patients with DRF while monitoring for hematotoxicity, especially thrombocytopenia and anemia.

Clinical phase III trials have reported a 2.4% incidence of thrombocytopenia in patients receiving linezolid therapy [67]. In our meta-analysis, the incidence of thrombocytopenia in patients with and without DRF ranged between 28.9 and 78.6% (except for the study by Hiraki et al. [25]) and 10.5 and 42.9%, respectively, which were higher than those previously reported. Nearly all the patients included in this meta-analysis were from Asian countries, such as Japan, China, and Korea, and had lower body weights than those of individuals from Western countries. Previously, lower body weight was considered a risk factor for thrombocytopenia [23]. Generally, linezolid was administered twice daily (600 mg × 2) and the dose was not adjusted by body weight. A comparison of the median weights among the groups that received linezolid treatment showed that the median weight was 80 kg when the AUC was 95.2 mg × h/L [53] and 58.3 kg when the AUC was 291.6 mg × h/L [45]. The difference in AUC values may be accounted for by the difference in the dose per body weight. Additionally, advanced age [68] and the duration of administration [69] are also considered risk factors; therefore, this difference in the patients’ backgrounds may explain the higher incidence of hematotoxicity.

A major reason for the higher incidence of thrombocytopenia in patients with DRF than in patients without DRF is the delayed excretion of linezolid and increased blood linezolid concentrations. Approximately 30% of linezolid is excreted by the kidneys of patients with normal renal function [70]. Furthermore, Matsumoto et al. evaluated the clearance of linezolid with renal function and reported a correlation between linezolid and creatinine clearance or blood urea nitrogen [69]. Therefore, we hypothesized that linezolid overexposure or higher C_min_ is associated with decreased renal function [59, 71].

In this meta-analysis, no significant differences were observed in the incidence of pancytopenia. This result does not indicate the absence of a relationship between DRF and the incidence of pancytopenia, as the number of cases included in the systematic review was notably smaller than that of thrombocytopenia. In addition, many studies have focused on thrombocytopenia, which occurs most frequently among the different forms of hematotoxicity (Sheldon et al. 2003 [5];). Therefore, it might have been easier to identify significant differences in thrombocytopenia. If more studies on pancytopenia are published in the future, significant differences in the incidence of pancytopenia will be found.

The incidence of thrombocytopenia was higher when the C_min_ of linezolid exceeded 6–7 μg/mL (Tables 4 and 5). Previous studies revealed the efficacy and safety ranges of linezolid trough values as 2–8 μg/mL [15, 16, 62, 72], 3.6–8.2 μg/mL [61], and 2–7 μg/mL [73]. In this study, we conducted a systematic review of the incidence of thrombocytopenia and C_min_ in children and adults, as determined by the extracted C_min_ threshold; the incidence of thrombocytopenia was higher when the C_min_ exceeded 6–7 μg/mL. However, this systematic review could not determine the clinically relevant threshold of linezolid in terms of the AUC (Tables 2 and 3). Matsumoto et al. reported a strong correlation between AUC and trough concentrations [61]. Only four studies reported the AUC values for patients with thrombocytopenia in this study.

Further studies are required to determine the target AUC that correlates with thrombocytopenia. However, it is difficult to measure the AUC in clinical settings; therefore, C_min_ may be a surrogate index of AUC in clinical practice. Consequently, we believe that therapeutic drug monitoring should be performed for linezolid administration from the perspective of safety and that the dose should be controlled to achieve a target trough value of < 6–7 μg/mL.

The previous meta-analysis showed that impaired renal function was associated with an increased risk of linezolid-induced thrombocytopenia [74]. Based on this knowledge, finding an association between hematotoxicity and patients with DRF, we classified hematotoxicity and performed a subgroup analysis, which showed that thrombocytopenia and anemia were significantly higher in patients with DRF than in those without DRF. We also conducted a systematic review and determined that hematotoxicity was higher when C_min_ exceeded 6–7 μg/mL. This finding is a strength of the current study. To our knowledge, this study is the first systematic review to explore the association of C_min_ with linezolid safety. This result may serve as an indication for the implementation of therapeutic drug monitoring and provide insights for further clinical trials.

This study had several limitations. First, most of the analyzed studies were observational studies. Therefore, the patient characteristics and study designs contained various types of bias, hindering their results’ generalizability. Second, the definitions of thrombocytopenia were different in these studies. Third, the estimation method of AUC differed in each study. This might have led to a misunderstanding of our results. However, this analysis did not clarify the target AUC due to the limited number of studies.

## Conclusion

Decreased renal function correlates with an increased risk of thrombocytopenia and anemia due to overexposure. To maximize the efficacy and minimize the toxicity of linezolid, therapeutic drug monitoring should be recommended, using evidence-based thresholds in patients on long-term linezolid treatment or in patients with DRF.

## Supplementary Information

**Additional file 1: Fig. S1.** Assessment of the risks of bias for studies included in meta-analysis. **Fig. S2.** Assessment of the risks of bias for studies included in systematic review.

## Data Availability

The datasets used and/or analyzed during the current study are available from the corresponding author upon reasonable request.

## Abbreviations

DRF: Decreased renal function
ORs: Odds ratios
CIs: Confidence intervals
PK: Pharmacokinetics
AUC: Area under the concentration curve
C_min_: Minimum blood plasma concentration
