# Supplementary material for: Safety of linezolid in patients with decreased renal function and trough monitoring: a systematic review and meta-analysis
Source: BMC Pharmacol Toxicol. 2022 Nov 30;23:89. doi: 10.1186/s40360-022-00628-9 (PMC9714190; doi:10.1186/s40360-022-00628-9)
Supplement: Supplementary file 1 — Additional file 1: Fig. S1. Assessment of the risks of bias for studies included in meta-analysis. Fig. S2. Assessment of the risks of bias for studies included in systematic review. [file 40360_2022_628_MOESM1_ESM.pptx]

## Slide 1
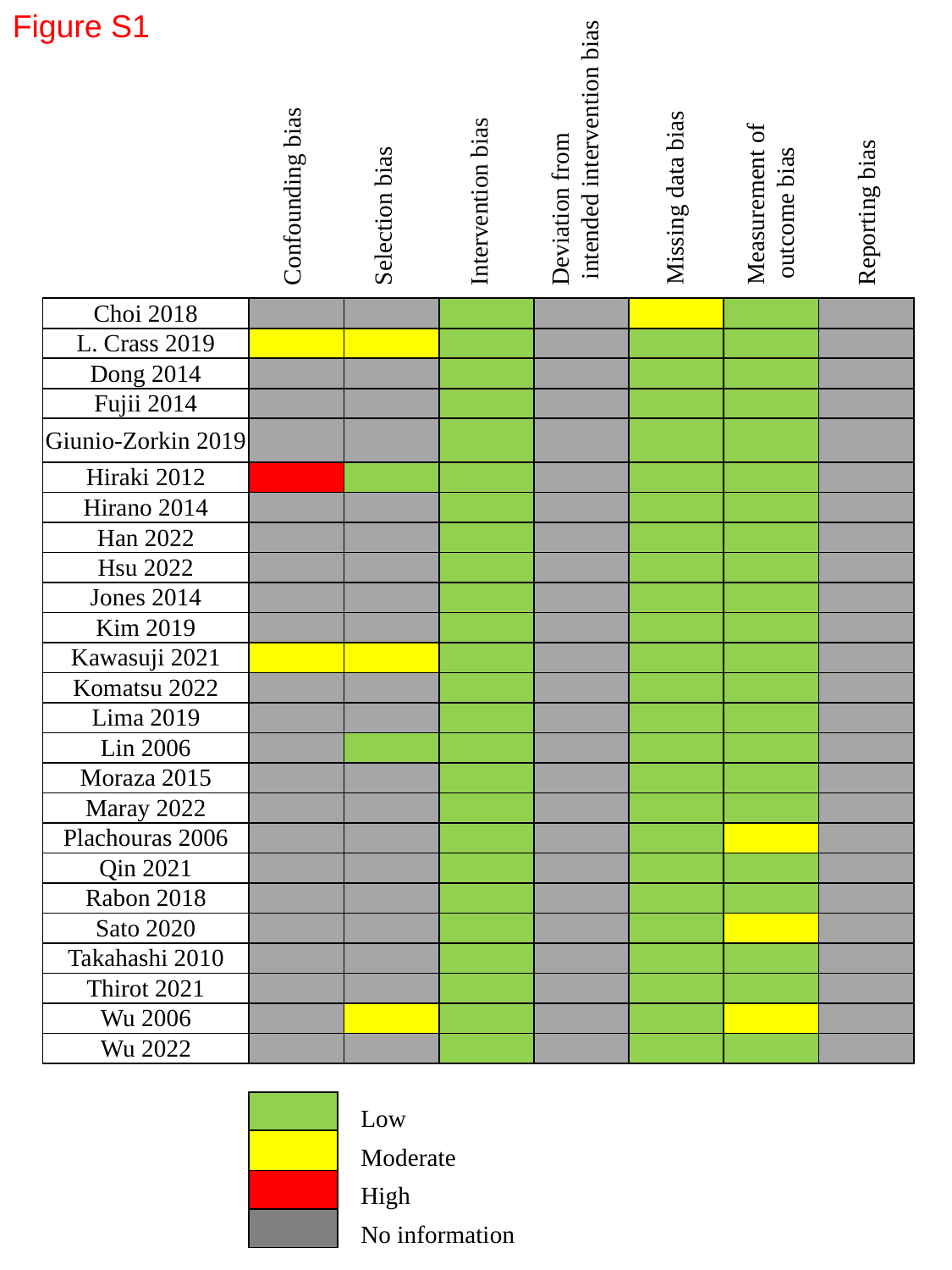

Figure S1
Deviation from
 intended intervention bias
Measurement of
 outcome bias
Confounding bias
Missing data bias
Intervention bias
Reporting bias
Selection bias
| Choi 2018 | | | | | | | |
| --- | --- | --- | --- | --- | --- | --- | --- |
| L. Crass 2019 | | | | | | | |
| Dong 2014 | | | | | | | |
| Fujii 2014 | | | | | | | |
| Giunio-Zorkin 2019 | | | | | | | |
| Hiraki 2012 | | | | | | | |
| Hirano 2014 | | | | | | | |
| Han 2022 | | | | | | | |
| Hsu 2022 | | | | | | | |
| Jones 2014 | | | | | | | |
| Kim 2019 | | | | | | | |
| Kawasuji 2021 | | | | | | | |
| Komatsu 2022 | | | | | | | |
| Lima 2019 | | | | | | | |
| Lin 2006 | | | | | | | |
| Moraza 2015 | | | | | | | |
| Maray 2022 | | | | | | | |
| Plachouras 2006 | | | | | | | |
| Qin 2021 | | | | | | | |
| Rabon 2018 | | | | | | | |
| Sato 2020 | | | | | | | |
| Takahashi 2010 | | | | | | | |
| Thirot 2021 | | | | | | | |
| Wu 2006 | | | | | | | |
| Wu 2022 | | | | | | | |
Low
Moderate
High
No information
| |
| --- |
| |
| |
| |

## Slide 2
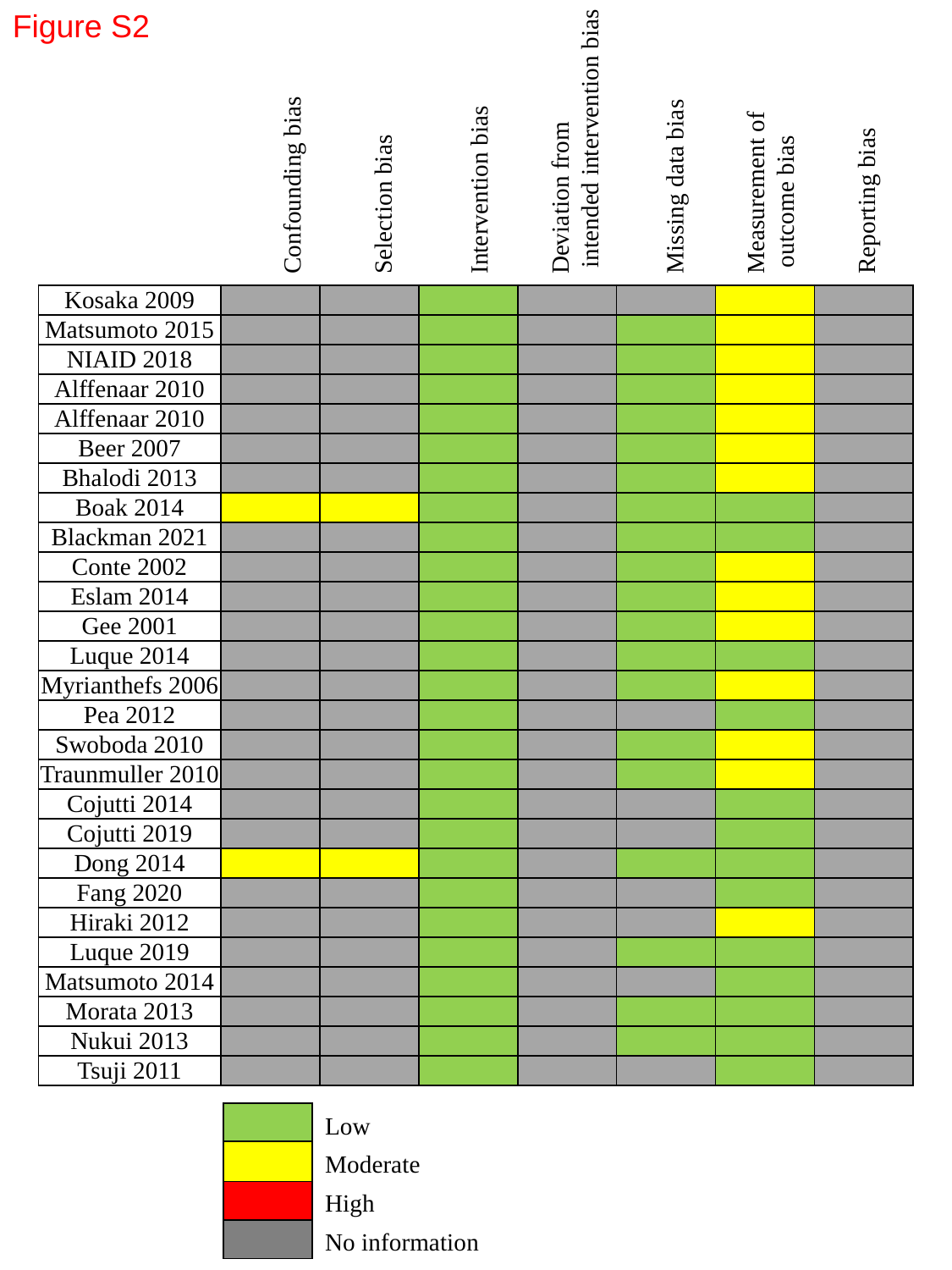

Figure S2
Deviation from
 intended intervention bias
Measurement of
 outcome bias
Confounding bias
Missing data bias
Intervention bias
Reporting bias
Selection bias
| Kosaka 2009 | | | | | | | |
| --- | --- | --- | --- | --- | --- | --- | --- |
| Matsumoto 2015 | | | | | | | |
| NIAID 2018 | | | | | | | |
| Alffenaar 2010 | | | | | | | |
| Alffenaar 2010 | | | | | | | |
| Beer 2007 | | | | | | | |
| Bhalodi 2013 | | | | | | | |
| Boak 2014 | | | | | | | |
| Blackman 2021 | | | | | | | |
| Conte 2002 | | | | | | | |
| Eslam 2014 | | | | | | | |
| Gee 2001 | | | | | | | |
| Luque 2014 | | | | | | | |
| Myrianthefs 2006 | | | | | | | |
| Pea 2012 | | | | | | | |
| Swoboda 2010 | | | | | | | |
| Traunmuller 2010 | | | | | | | |
| Cojutti 2014 | | | | | | | |
| Cojutti 2019 | | | | | | | |
| Dong 2014 | | | | | | | |
| Fang 2020 | | | | | | | |
| Hiraki 2012 | | | | | | | |
| Luque 2019 | | | | | | | |
| Matsumoto 2014 | | | | | | | |
| Morata 2013 | | | | | | | |
| Nukui 2013 | | | | | | | |
| Tsuji 2011 | | | | | | | |
Low
Moderate
High
No information
| |
| --- |
| |
| |
| |
